# Beyond Audio-Video Telehealth: Perspective on the Current State and Future Directions of Digital Neurological Care in the United States

**DOI:** 10.2196/46736

**Published:** 2024-04-11

**Authors:** Benjamin R Kummer, Neil A Busis

**Affiliations:** 1 Department of Neurology Icahn School of Medicine at Mount Sinai New York, NY United States; 2 Clinical Neuro-informatics Program Icahn School of Medicine at Mount Sinai New York, NY United States; 3 Windreich Department of Artificial Intelligence and Human Health Icahn School of Medicine at Mount Sinai New York, NY United States; 4 Department of Neurology New York University Grossman School of Medicine New York, NY United States

**Keywords:** asynchronous telehealth, chronic condition management, COVID-19, digital health, digital neurology, eConsult, endemic, interprofessional consultation, neurological apps, neurological care, neurology, principal care management, remote patient monitoring, technology, telehealth legislation, telehealth, teleneurology

## Abstract

**Background:**

The COVID-19 pandemic transformed neurological care by both requiring digital health modalities to reach patients and profoundly lowering barriers to digital health adoption. This combination of factors has given rise to a distinctive, emerging care model in neurology characterized by new technologies, care arrangements, and uncertainties. As the pandemic transitions to an endemic, there is a need to characterize the current and future states of this unique period in neurology.

**Objective:**

We sought to describe the current state of the pandemic- and postpandemic-related changes in neurological care and offer a view of the possible future directions of the field.

**Methods:**

We reviewed several themes across the “new digital normal” in neurology, including trends in technology adoption, barriers to technology access, newly available telehealth services, unresolved questions, and an outlook on the future of digital neurology.

**Results:**

In this new era of neurological care, we emphasize that synchronous audio-video telehealth remains the predominant form of digital interaction between neurologists and patients, mainly due to pandemic-related regulatory changes and the preexisting, steady adoption of video platforms in the prepandemic era. We also identify a persistent digital divide, with audio-only telehealth remaining a necessity for preserving care access. Asynchronous telehealth methods and services, including care coordination, interprofessional consultations, remote patient monitoring, and teletreatment are becoming increasingly important for neurological care. Finally, we identify several unanswered questions regarding the future of this “new normal,” including the lasting effects of emergency regulatory changes, the value proposition of telehealth, the future of telehealth reimbursement in neurology, as well as privacy considerations and trade-offs in asynchronous neurological care models.

**Conclusions:**

The COVID-19 pandemic has ushered in an era of digital adoption and innovation in neurological care, characterized by novel care models, services, and technologies, as well as numerous unresolved questions regarding the future.

## Introduction

The COVID-19 public health emergency significantly accelerated the adoption of digital technology in neurological care [1] and established synchronous and asynchronous telehealth as widely accepted care modalities across multiple subspecialties of neurology [2-7]. While historic, this acceleration also built upon the momentum generated by 2 decades of growing digital technology and service adoption in neurology. This momentum included the advent of telestroke [8], the establishment of video telehealth care programs in rural areas of the United States [9], and the growing use of smartphones and wearable devices in neurological care paradigms and research [10]. Furthermore, broad telehealth trends that led up to the COVID-19 pandemic, such as the shift of telehealth from acute to chronic neurological conditions, migration of care toward mobile device platforms, and increasing focus on patient convenience and value [11], also likely facilitated the shift to digital and web-based neurological care in 2020.

Approximately 3 years after the start of the COVID-19 pandemic in the United States, the field of neurology has transitioned to a new digital environment, encompassing new and emerging care models and services, novel technologies, as well as new and persistent challenges and open questions. While this new digital landscape is wide-ranging, complex, and often subject to rapid changes, a comprehensive appraisal of the current state of care can nonetheless be helpful in establishing policy priorities and identifying opportunities to improve access to digital technologies for patients with neurological conditions. In this review, we sought to describe the digital state of neurology care in the COVID-19 and post–COVID-19 eras, placing emphasis on dominant forms of digital neurological care, emerging technology trends and technology-enabled digital neurology services, barriers to access to digital care, telehealth in education, as well as ongoing challenges and uncertainties facing the future.

## Themes

### Video Telehealth Is the New Dominant Digital Care Modality

#### Comparisons Between Pre–COVID-19 and Late Pandemic Use

The COVID-19 era saw synchronous audio-video (or simply “video”) telehealth fundamentally shift away from a novelty technology garnering little interest among most practicing neurologists to an acceptable alternative to in-person face-to-face encounters and other traditional neurological care modalities for patients and providers [12]. In late 2021, the use of video telehealth in multiple medical specialties remained approximately 38 times higher in the United States than before the onset of the pandemic and comprised 13% of neurology outpatient visit claims nationwide [13]. On the health system level, the use may be even higher, with certain rural health systems recently noting that nearly 35% of ambulatory neurology visits were conducted through telehealth. For many neurologists nationwide, synchronous video telehealth remains the preferred mode of telehealth delivery, followed by audio-only telehealth [14]. Compared to the relatively infrequent use of video telehealth in neurology before 2020, these findings all underline the important place video telehealth now occupies in modern neurological care.

#### Factors Driving the Rise and Predominance of Video

Insurance payment incentives were important in driving video telehealth’s initial rise to prominence in neurology during the pandemic, especially in the United States. In declaring the COVID-19 public health emergency, the Centers for Medicare and Medicaid Services (CMS), the nation’s largest insurance payor, suspended multiple geographic restrictions for video telehealth insurance reimbursement that had previously limited patients from being evaluated over video telehealth in their homes and outside of designated rural areas, effectively limiting uptake and contributing to the “novelty” status of video telehealth before the pandemic [15]. The lifting of such restrictions early on in the pandemic and their continuing suspension in later stages of the pandemic have incentivized patients, providers, practices, and health care systems to widely use video telehealth.

Additional factors that have contributed to the continued dominance of video telehealth in neurology include high and steadily increasing rates of smartphone ownership across the world [16] and the liberal allowance of several platforms for telehealth, particularly in the United States. More specifically, enforcement discretion of HIPAA (Health Insurance Portability Accountability Act) regulations by the US Department of Health and Human Services during the public health emergency allowed non–HIPAA-compliant technology platforms to be widely used for video telehealth purposes [17].

Patients and neurologists have reported positive experiences with video telehealth, which have likely preserved telehealth’s dominance as a digital offering in our current era. Video telehealth is perceived as convenient [18,19] and rated as highly satisfactory among patients [2,20]. Similarly, notwithstanding some reports suggesting that providers have had greater challenges than patients with video telehealth encounters [2], neurologists have generally found satisfaction, positive experience [21,22], and effectiveness [23] with video telehealth visits.

#### Elements of the Neurological Examination

Although the feasibility and accuracy of a detailed, video-based neurological examination have been the subject of debate among the neurological community, the pandemic era mandated the need for remote neurological examinations and accelerated the adoption of additional examination methodologies for performing the digital neurological examination beyond video technology. These phenomena build upon previous work demonstrating that video-based neurological examinations can accurately be used to administer standardized disease-specific examinations, such as the Unified Parkinson Disease Rating Scale (UPDRS) for Parkinson disease [24], the Unified Huntington Disease Rating Scale [25], or the Montreal Cognitive Assessment in individuals with movement disorders [26]. Additional examples include digital versions of the Expanded Disability Severity Scale in multiple sclerosis [27], the Multiple Sclerosis Performance Test [28], or the Myasthenia Gravis TeleScore [29].

While recent work has suggested not only that many elements of the neurological examination could be completed over video telehealth, additional studies have suggested that patients themselves may be assessed through functional evaluation (eg, performing exercises or shifting from sitting to standing position), serve as their own examiners, as well as use household items such as flashlights, toothpicks, or weights to aid neurological assessments [30,31]. More importantly, most elements that are most useful for neurological decision-making can be performed over a video connection [23].

Despite this, several elements of the neurological examination remain challenging to routinely perform over video telehealth, such as fundoscopy, vestibular testing, visual field examination, and muscle tone. Among these elements, televestibular and fundoscopy assessment technologies currently exist but typically require additional hardware beyond video-enabled smartphones, thereby creating persistent barriers to use for most patients and providers. Although these shortcomings do exist, they nonetheless represent fertile ground for future technological innovations to address the objective of completing entirely digital neurological examinations. Indeed, neurologist surveys suggest that devices to perform gait, sensory, fundoscopic, oculomotor, and strength assessments are highly desirable to complement the video examination [32].

Perceptions of the adequacy of the digital neurological examination may also vary according to subspecialty. In a recent survey of academic neurologists, neuromuscular specialists expressed dissatisfaction with performing the neurological examination over video, mainly due to an inability to assess reflexes and tone. By contrast, movement disorder specialists expressed concern over inadequate internet bandwidth for bradykinesia assessments as well as unwieldy camera angles that precluded in-depth evaluation of gait [33].

While these perceptions express some sense of dissatisfaction, they nonetheless reflect that different neurological subspecialties tend to emphasize different components of the neurological examination (and, by extension, the remote neurological examination) more than others. Accordingly, numerous subspecialty-oriented teleneurology examination guides have been developed since the onset of the COVID-19 pandemic, which are now available through multiple web sources, including professional society web pages [34].

These guides emphasize examination elements that differ according to subspecialty. For instance, neuromuscular examination guides suggest using validated scales such as the Myasthenia Gravis Activities of Daily Living or the Revised Amyotrophic Lateral Sclerosis Functional Rating scales, assessing upper extremity tone by holding the patient’s arms out and shaking them to assess for rigidity, determining motor strength by observing limb movement against gravity, and evaluating plantar responses by asking the patient to stimulate the plantar surface of their feet with a pen [35]. By contrast, guides for neurovestibular or neuro-ophthalmic disorders tend to emphasize the oculomotor examination and vestibular or visual field testing [36].

### Evidence Supporting Teleneurology

In the decade leading up to the COVID-19 pandemic, a multitude of studies had already investigated the quality impacts of specific teleneurology care, including user satisfaction and diagnostic accuracy, as well as impacts on clinical outcomes, costs, and care access across multiple neurological conditions encompassing dementia, multiple sclerosis, movement disorders, headache disorders, inpatient neurology, traumatic brain injury, neuromuscular disorders, and epilepsy (Table 1). Randomized controlled and inferiority trial evidence generally suggests that teleneurology is associated with positive impacts on clinical outcomes, diagnostic accuracy, and physician or patient satisfaction. Studies carried out in the post–COVID-19 era have demonstrated similar findings with respect to satisfaction [37]. Improvements in cost-savings and care access were noted in mainly small or nonrandomized studies, although there were notably absent studies suggesting the latter in dementia, headache, multiple sclerosis, and neuromuscular disorders (Table 1) [38].

At the time of writing, nearly 50 US institution–sponsored telehealth trials in prevalent neurological disorders, including Parkinson disease, stroke, multiple sclerosis, epilepsy, Alzheimer dementia, and headache disorders, are either active or currently recruiting participants. Although a small minority of these initiatives are not yet recruiting, these studies include both observational and interventional trials to evaluate a range of outcomes, including but not limited to feasibility, comparative effectiveness, cost-effectiveness, and safety measures (Multimedia Appendix 1).

**Table 1:** Summary of available data across multiple quality measures of teleneurology by specialty. The table represents extant evidence on telehealth in neurology as of early 2020. Reproduced with permission from Wolters Kluwer from Hatcher-Martin et al [38].

	Patient and physician satisfaction	Improved access to care	Diagnostic accuracy	Improved outcomes	Cost savings (patient and health system use)
Concussion or traumatic brain injury	+^a^	+	++^b^	++	+
Dementia	++	—^c^	++	+	+
Epilepsy	+	+	—	++	+
Headache	++	—	++	++	+
Movement disorders	++	+	++	++	+
Multiple sclerosis	++	—	++	++	+
Neuromuscular	++	—	—	+	+
Inpatient general neurology	—	+	+	+	+

^a^+: small case series, indirect measurement.

^b^++: randomized controlled trial or inferiority trial, direct measure.

^c^No studies.

### Factors Limiting Digital Neurology Uptake

#### Persistent, Widespread Disparities and Barriers

Several digital and socioeconomic inequalities in the US health care system clearly preceded the COVID-19 crisis that persisted throughout the early and late phases of the pandemic and profoundly impacted the adoption of digital care modalities during the public health emergency. Indeed, telehealth was less readily adopted among low-income, minority, non–English-speaking, and governmentally insured neurological populations during the early and middle stages of the pandemic [4,39,40], and access to audio-video telehealth has continued to demonstrate limited uptake among Black and governmentally insured populations in later pandemic stages [41].

Defined as “the gap existing between individuals who have access to modern information and communication technology and those who lack access” [42], the “digital divide” has been cited as a primary driving factor for asymmetrical digital neurology service adoption in the COVID-19 era. This perception has also persisted among providers. More than 2 years after the beginning of the COVID-19 pandemic, this “digital divide” continues to serve as the largest barrier to offering telehealth services among US providers [14]. Possible causes driving these asymmetries may include digital literacy, a lack of non–English-language interfaces, the prohibitive economics of steady digital access, limited access to broadband internet, inadequate cellular data plan coverages, and potentially cultural factors.

It remains important to note that many of the disparities that have been observed in the uptake of telehealth in neurology are not unique to digitally enabled care platforms. Rather, they tend to closely mirror existing sociodemographic disparities in access to neurological care that have been long observed in “nontelehealth” neurological care. Indeed, socioecological factors have been identified by numerous stakeholders as driving the vast majority of health disparities in neurological care [43]. Analyses of specific neurological conditions also reflect sociodemographic disparities in care. For example, Black and Hispanic patients are less likely to see outpatient neurologists across a range of neurological disorders, including headache disorders, Parkinson disease, stroke, and epilepsy [44]. Similarly, Black patients have lower odds of receiving thrombolytic therapy for acute ischemic stroke nationwide than White patients. Rural patients have similarly decreased odds compared to urban patients, as do patients living in ZIP codes with median incomes under US $64,000 in comparison to those living in wealthier ZIP codes [45]. A number of additional analyses have emphasized racial or sex-based disparities in multiple neurological disorders and treatments, including deep brain stimulation and general treatment for Parkinson disease, temporal lobe resection for medication-refractory epilepsy, evaluation and management of neuro-oncologic conditions, and treatment of acute stroke [46-51].

#### The Critical Importance of Audio-Only Telehealth

In light of the digital divide and asymmetric digital neurology adoption, audio-only services remain centrally important to the new digital normal in neurology. Synchronous, audio-only telehealth has played an important role as an alternative to synchronous audio-visual telehealth since the outset of the COVID-19 pandemic in 2020. This role has persisted through multiple phases of the pandemic, particularly for populations lacking regular access to broadband internet and cellular data connectivity, including older people, disabled people, or socially disadvantaged groups among both nonneurological [52,53] and neurological populations [39,40,54].

Although single-center evidence suggests that usage of telephone services may have steadily decreased in academic centers in later stages of the pandemic [55], a primary driving force toward use of audio-only telehealth services throughout the pandemic was CMS’ decision in March 2020 to temporarily add American Medical Association (AMA) Current Procedural Terminology (CPT) telephone-only evaluation and management billing codes to a list of billable telehealth services for the duration of the public health emergency [15].

Several factors underscore the important role of audio-only telehealth currently plays and will likely continue to play in care delivery during the pandemic era and beyond. At the time of writing, the US government has upheld the declaration of the COVID-19 public health emergency, thereby guaranteeing that telephone services will continue to be treated as billable telehealth services through the calendar year 2023. Furthermore, audio-only services continue to provide a crucial access point to health care. Indeed, a significant proportion of providers continue to use audio-only telehealth, with many reporting this to be second only to synchronous audio-video telehealth [14]. Recognizing the importance of audio-only telehealth, professional societies such as the American Academy of Neurology have called on CMS and the US Congress to make reimbursement rates for audio-only services permanent after the cessation of the federally declared COVID-19 public health emergency.

### The Increasing Importance of Asynchronous Telehealth

#### Asynchronous Teleneurology

Synchronous telehealth currently occupies a central position in the universe of today’s available complement of digital neurology services. By comparison, asynchronous telehealth, in which geographically disparate participants are separated by time as well as location, remains poorly used. However, it is important to the growing importance of asynchronous telehealth as part of the “new digital normal” in neurology. At the most basic level, this form of telehealth includes well-established modes of digital communication, such as email and SMS text message, but can range to more complex technological implementations. From a functional perspective, asynchronous telehealth in neurology can be organized into 4 general categories: remote diagnostic services (telemonitoring), remote delivery of neurological treatments (teletreatment) [56], electronic interprofessional consultations, and care coordination.

The pandemic era has seen a number of new billable clinical activities emerge in the United States that have facilitated the rising importance of asynchronous care services in neurology. These services include remote patient (also termed “physiologic”) and therapeutic monitoring, digital check-ins, digital evaluation and management, principal care management (PCM), and interprofessional consultations. In addition to these billable services, these activities also substantiate a growing trend in digital neurology in which centralized, inconvenient, and synchronous care models are progressively shifting toward distributed, asynchronous models that prioritize patient convenience and access [10]. The onset of the COVID-19 pandemic in early 2020 accelerated this shift by expanding the adoption of asynchronous services as well as synchronous ones [57,58].

#### Telemonitoring

Neurological telemonitoring now encompasses a wide range of clinical services. A commonly encountered form of telemonitoring includes smartphone apps or electronic health record (EHR) questionnaires that receive patient-centered symptoms, validated clinical scales, or medication compliance information that is then transmitted electronically to a care team with the purpose of establishing a diagnosis or monitoring responses to treatment [59]. Examples of such apps abound in neurology, which comprises many chronic, polyphasic disorders such as migraine [60-62], multiple sclerosis [63,64], epilepsy [65], and Parkinson disease [66], among others.

Telemonitoring also includes “store and forward” services, in which a patient transmits clinical image information such as digital image, recorded audio, or video to a treating provider team for asynchronous review. A particularly useful application of store-and-forward in neurology is the diagnosis of paroxysmal neurological events, such as seizure-like episodes [67], as well as a close review of dynamic neurological examination findings in Parkinson disease [68-70].

Remote patient monitoring (RPM), an already well-established form of telemonitoring in nonneurological conditions such as congestive heart failure, chronic obstructive pulmonary disease, and diabetes, occupies an increasingly important position in the care delivery to patients with neurological disorders. Similar to nonneurological applications, neurological RPM uses sensor-containing patient wearable devices, occasionally paired with mobile app platforms, to record and transmit continuous or near-continuous physiological information to care providers for review and medical decision-making over a secure internet connection [71]. In neurology specifically, the growing importance of telemonitoring capitalizes on the growing understanding that episodic patient assessments often provide incomplete and sometimes inaccurate assessments of patients’ clinical and functional status [10].

However, neurological RPM notably differs in data acquisition and transformation techniques from its nonneurological counterpart. Because most neurological disorders rely on a combination of qualitative radiographic or clinical examination findings to establish a diagnosis or inform management rather than laboratory or vital sign information, neurological RPM generally uses raw data from limb accelerometer and gyroscope sensors to extrapolate meaningful “digital biomarkers” such as gait, arm swing, step count, falls, examination findings, or abnormal movements. This is in contrast to nonneurological RPM, where sensors directly measure clinically relevant biomarkers such as blood pressure, blood glucose, or oxygen saturation, for example [72,73].

Notable areas of RPM application to neurology include disorders with prominent motor and gait features such as multiple sclerosis [74] and movement disorders [75-78]. In addition to demonstrating feasibility and acceptability, RPM has potentially identified novel digital biomarkers. One notable example is the daily step count, which is associated with functional status decline in patients with multiple sclerosis [74] and incident dementia [79]. While these RPM approaches are not yet established as standard-of-care, they are being used increasingly in clinical and research applications with an understanding that further work is required to better grasp the implications of collecting and transmitting this information [56].

Important to note are the few instances of fully integrated, scaled neurology RPM programs in health care systems in the United States as well as the relatively underused nature of these services by neurologists. Nationwide analyses of US Medicare claims data suggest that neurologists comprise a very small proportion of RPM-billing providers [80,81]. Interestingly, analysis of nationwide commercial claims data shows that only 14% of the nearly 17,000 RPM encounters billed by physicians to commercial payers for neurological disorders between 2019 and 2021 were billed by neurologists, compared to 57% that were billed by family medicine, pulmonary, and internal medicine providers combined. Moreover, nearly 90% of these encounters were billed for sleep-wake disorders, with approximately 2% billed for common neurological conditions such as cerebrovascular disorders, movement disorders, epilepsy, migraine disorders, and polyneuropathies combined (B Kummer et al, unpublished data, 2023). These data suggest that despite its promise, RPM is underused by neurologists for neurological conditions, particularly those that constitute relatively straightforward clinical use cases, such as blood pressure monitoring after stroke, or step counting in multiple sclerosis, movement disorders, or neuropathies.

While billing activity reflects a limited dimension of RPM use, the reasons for these findings could be that few Food and Drug Administration–approved devices (a requirement for billing new RPM codes issued after 2019) for monitoring physiologic signals in neurological conditions currently exist. Alternatively, high variability in the quality and availability of commercial wearables and sensors may explain RPM underuse by neurologists. Finally, the lack of integration of many RPM solutions into EHR systems is likely a contributing factor that has been identified as an important barrier to the adoption of RPM services into real-world clinical settings across a spectrum of medical specialties [82].

#### Teletreatment

Neurological teletreatment is now widely available for the management of headache, epilepsy, and movement disorders. A notable category of teletreatment options comprises stimulator devices that deliver focused electricity to selected nervous system regions [83], including vagal nerve stimulators, responsive neurostimulators, and deep brain stimulators, which have all found application in epileptic [84] and movement disorders [85]. In migraine and other headache disorders, analogous devices include peripheral stimulator devices targeting the supraorbital, occipital, or sphenopalatine ganglion [86]. Many of these devices can be remotely programmed by a provider as well as collect and relay neurophysiologic data back to care teams for treatment decisions. Furthermore, device programming parameters can potentially be integrated into EHR systems to provide a snapshot of the patient’s clinical status.

Some authors consider technology, per se, to constitute treatment [87] and therefore represent an additional subcategory of teletreatment. Under this conceptual framework, mobile health apps that are capable of various monitoring and diary functions may be thought of as treatment in and of themselves. One notable application of “technology as treatment” includes headache disorders, where symptom diaries may provide insight into disease processes and inform treatment or guide complementary and integrative therapies that modulate stress levels and pain perception [59].

#### Care Coordination

In response to the rising prevalence of chronic conditions and their significant associated costs in the United States, CMS has developed billable care management and coordination services in the second decade of the 21st century that make extensive use of asynchronous telehealth interactions and represent another increasingly important example of asynchronous teleneurology in the COVID-19 era. These services are best exemplified by chronic care management (CCM; introduced in 2015), which supports care management of multiple chronic conditions, and PCM (introduced in 2022) for the management of a single complex condition. These services incentivize an integrated, team-based approach to chronic condition management by bundling care coordination, care planning, and condition-focused goal setting into an overarching care management activity that is primarily furnished through non–face-to-face encounters. Both PCM and CCM allow care teams to interact with patients asynchronously, using the technology platform of their choice. Furthermore, CCM specifically includes care monitoring in the definition of billable service, thereby allowing the use of RPM and remote therapeutic monitoring.

In addition to CCM and PCM, coordination of care can be performed through asynchronous patient portal communications between patients and providers. These communications dramatically increased with the onset of the COVID-19 pandemic [88], potentially as a result of increased video telehealth adoption and the absence of office-based follow-up arrangements. In addition to care coordination, the potential for completing true evaluation and management of new medical problems over patient portals led to the introduction of new digital evaluation and management services (or “e-visits”) in 2020 as billable codes (CPT codes 99421-99423 and Healthcare Common Procedure Coding System codes G2061-G2063). While several US health care institutions in the United States have successfully implemented billing for e-visits and increased the volume of these services [89], some of these implementations were accompanied by decreases in the use of portal messaging and suggested that few portal messages were truly billable as e-visits, arguing that these services have not lessened the cognitive overload imposed by significant increases in patient portal messaging [90,91].

#### Interprofessional Consultations

Although much of neurological telehealth refers to patient-provider interactions, consultations between providers remain an important area of digital care in neurology. Telephone calls between providers and synchronous video teleneurology consultations have existed for decades, with telestroke constituting perhaps the most widely known example of the latter [8]. Despite this, a growing number of interprofessional neurology consultations are now performed asynchronously and have been successfully implemented in headache and neuro-ophthalmic conditions, leveraging electronic forms of communication such as email, clinical notes, or direct verbal communication over the telephone to requesting providers [92-95]. Although discussion of recommendations with the requesting provider may be a synchronous interaction, the bulk of the service is provided asynchronously.

Aside from the application of interprofessional consultations to specific neurological conditions, some notable use cases for this emerging service include improving access to neurological expertise in the setting of worldwide neurologist shortages [93,96], limiting personal exposures to hospitalized patients with diseases carrying significant infectious risk such as COVID-19, or improving the ability to evaluate and manage common neurological problems among nonneurologists [95]. To incentivize this activity, in a manner similar to CCM and PCM, CMS has delineated billable interprofessional consultation services, for which a discrete number of acceptable billing codes have been developed [97].

### The Future of Digital Neurology

The future of digital neurology can be organized into 3 broad areas: new information processing methods, new data types, and the provision of care through new modes of interaction. New processing methods are likely to include artificial intelligence (AI) processes that automate the detection of clinically meaningful information (assistive AI), analyze automatically collected information (augmentative AI), or analyze and draw independent conclusions from providers (autonomous AI) [98]. While assistive and augmentative AI is already in use within individual disease states, including stroke [99], Parkinson disease [68-70], or epilepsy [100,101], augmentative AI remains the least widely represented approach.

However, AI processes will probably not evolve to replace providers or medical decision-making but rather automate simple processes to allow providers greater bandwidth to tackle an increasingly complex array of neurological disorders [102].

In addition to the growing role of AI, multilayer synthesis, or “phenotyping,” of complex data streams is likely to become more common as the use of physiological, structured EHR, textual, and other data streams grows in neurological disorders [103]. This phenotyping may be used to serve multiple objectives, including the automation of standardized clinical assessments in key disorders such as the National Institutes of Health Stroke Scale or the UPDRS, the characterization of clinically meaningful disorder manifestations or outcomes, or the identification of novel disease subpopulations.

The future of digital neurology will also likely entail the exchange of novel data types, including videos of neurological events, examinations, and phenomenology, with or without AI assistance, as well as social network activity and geo-localization data to quantify patient “digital life space.” Treatment information, such as responses to individual therapies, adverse events, medication compliance, and symptom diaries, is likely to become increasingly common within the ongoing digitization of neurology. Additionally, as sensors become increasingly sophisticated and compact, RPM in neurological disorders will likely evolve to incorporate additional sensor streams such as magnetometry, skin galvanic responses, and other novel biomarkers into routine clinical care [103].

Finally, private companies and health system strategies’ shift toward convenience- and patient-oriented care journeys is likely to impact the manner in which patients with neurological conditions and providers interact. Semi- or fully automated chatbots, which are already widely available in the retail and banking industries, may eventually provide around-the-clock access for simple questions that do not require high-level clinical decision-making. Recent private-sector initiatives featuring on-demand, search-engine–based and technology-forward health care for large populations of patients [104-106] suggest that such “digital front doors” may become the primary method of locating neurological expertise and obtaining resources for patients with neurological disorders, rather than relying on referrals from providers and other traditional pathways.

### Unanswered Questions: a Look Toward the Future

#### The Telehealth Value Proposition

The value of telehealth and whether telehealth adequately attains desired health outcomes relative to the cost of care delivery [107,108], remains a largely open question across medical specialties. Although video telehealth is associated with significant patient and provider benefits, it has been shown to generally increase costs, with the exception of cases of eliminating long-distance travel [109]. More recently, a study investigating the value of telehealth in young adults with cancer overwhelmingly found that telehealth resulted in cost savings [110].

In contrast to the limited investigations of value in nonneurological conditions, modern telehealth for neurological care faces an uncertain future with respect to the question of value. Although the question chiefly concerns synchronous audio-video telehealth, which is arguably the most common digital neurology interaction today, the telehealth value question remains relevant to all forms of digital neurological care [14]. Outside of synchronous telestroke care, which has long been one of the clearest examples of telehealth value in neurology before the COVID-19 pandemic era [111,112], there remains a dearth of information regarding whether synchronous telehealth provides an acceptable value of care in noncerebrovascular neurological conditions. Large-scale, multicenter studies should address this specific question for synchronous audio-video as well as asynchronous forms of telehealth as applied to neurological disorders [108].

#### Governmental or Public Health Emergency Restrictions: the Future of Telehealth Reimbursement

By facilitating the adoption of various digital neurology modalities among providers and patients, the suspension of multiple telehealth reimbursement restrictions due to the COVID-19 public health emergency by the US federal government figures among the principal driving forces in catalyzing the widespread use of digital neurology services during the pandemic era [1]. At the time of writing, the public health emergency officially ended on May 11, 2023 [113], after which many suspended restrictions, such as CMS reimbursement for video telehealth visits irrespective of geographic locations, were extended into the end of 2024 [114]. However, many exemptions, including temporary reimbursement of specific telehealth services as category 3 codes and flexibilities involving controlled substance prescription over telehealth, among others, were extended only until the end of 2023. The rapidly changing flexibility landscape as well as the multiplicity of time frames create a complex matrix of different regulations that is often overwhelming and confusing to providers [115].

As opposed to federal-level restrictions, medical licensure and scope of practice continue to be regulated by individual US states, which restricts providers from delivering telehealth care to patients not located in states where the provider is licensed. To maximize patient access to telehealth care early in the COVID-19 pandemic, several US states loosened licensure requirements in order to allow out-of-state providers to easily obtain temporary licenses. However, since the end of the federal public health emergency, many states have rescinded these temporary flexibilities, with unclear impacts on telehealth use. It remains similarly unclear whether the Interstate Licensure Compact, an agreement signed by 37 US states and territories to simplify the licensure process for providers who wish to practice in multiple states [116], will positively impact the use of telehealth broadly speaking.

While the US Congress has introduced a bill to make several pandemic suspensions permanent [117], many specifics concerning the postpandemic regulatory landscape beyond 2024—and impacts on the long-term feasibility, viability, and adoption of digital modalities such as synchronous and asynchronous telehealth—remain unclear. As such, the rapidly approaching end of this extended period represents a significant source of uncertainty for the new digital normal.

#### Privacy Considerations of New Digital Interactions

Although privacy and security of personal health information for the purposes of medical care is strictly regulated by HIPAA in the United States, another important aspect of the new digital normal in neurology is the proliferation of digital technologies and services that collect and transmit personal health information but are not considered to be the provision of medical care or constitute a health care relationship under US federal law [118]. While this implies that they are not regulated under the purview of HIPAA, many of these technologies are nonetheless commonly used by providers and patients for the diagnosis and management of neurological conditions. Concerningly, mobile apps have been shown to disclose unauthorized personal health information outside of their end-user licensing agreements [119,120].

Patients using all forms of unregulated digital neurology services are therefore faced with a fundamental trade-off between collecting clinically meaningful information and infringing upon personal privacy. Sharing personal health information, even if knowingly, can potentially have undesired consequences. One particular venue in which this is evident is the growing phenomenon of employee wellness programs that collect physical activity and geospatial position information through wearable devices. These could disclose an employee’s actions during work unbeknownst to the wearer and potentially result in disciplinary action.

Open questions remain as to which venue is appropriate for regulating these issues. At the time of writing, in the United States, CMS and billing stakeholders such as the AMA have not taken any official stance against limiting the sharing of personal information on asynchronous teleneurology platforms, with most controls existing at the level of specific company data use policies and end user licensing agreements at the level of user acceptance.

#### The Future of Digital Neurology

During the COVID-19 public health emergency, digital neurology modalities clearly ensured safe access to neurological care for patients, resulting in significantly increased adoption and awareness of these tools among patients and providers. Asymmetric adoption of digital tools across different populations also cooccurred during the rapid rise in adoption, exposing the significant, persistent challenge facing the US health care system: access to specialty care [121]. Despite this, digital care modalities continue to demonstrate beneficial effects on care access and value [110,122-124] and carry even greater potential for the future of the health care system.

The “new digital normal”—within and outside of neurology—will realize this potential by reaching 3 critical milestones. The first is to shift the current digital operating framework, which places a significant focus on the range of available digital care solutions and their technical differences (eg, audio-only or audio-video and asynchronous or synchronous), to a structure emphasizing a tailored approach to digital care that combines “doses” of different technical solutions to individualized patient use cases.

The second will be to incorporate the rapidly growing array of AI technologies as complementary solutions in the current armamentarium of technical options targeting care access bottlenecks. By accelerating diagnosis recognition, automating clinical processes, and reducing provider cognitive overload, AI can effectively accelerate access to neurological expertise throughout the health care system. As such, this emerging set of technological innovations will likely prove itself to be a crucial complement to currently available digital tools.

The third milestone is creating a sustainable reimbursement framework that incentivizes providers to use digital tools. Efforts targeting this milestone are already underway at the time of writing and include the development of coding structures targeting clinical activities centered on specific technical solutions as well as classifying machine-performed clinical work [98,125].

## Conclusions

Contrasting with the temporary nature of the public health crisis itself, the COVID-19 pandemic has profoundly and indelibly altered the practice of neurology and medicine as a whole, ushering in an era of digital technology adoption and innovation characterized by novel care digital care models, services, and technologies. Despite the significant uncertainty and numerous unresolved questions facing this new digital normal in neurology, reverting to “prepandemic” technical solutions and care arrangements is failing to capitalize on one of the greatest opportunities to move medicine forward in the history of our species. It is crucial to consider the unprecedented scale and depth of digital health innovation that has occurred during this time [121] and the primordial importance of continued innovation in order to bring neurology and all specialties of medicine into the next phase of this “new digital normal.”

## Appendix

Multimedia Appendix 1 Summary of current (recruiting, active but not recruiting, and enrolling by invitation) US institution-sponsored clinical trials of telehealth in selected neurological disease that have launched since the start of the COVID19 pandemic in March 2020. Source: ClinicalTrials.gov; accessed December 7th, 2023.

## Abbreviations

AI: artificial intelligence
AMA: American Medical Association
CCM: chronic care management
CMS: Centers for Medicare and Medicaid Services
CPT: Current Procedural Terminology
EHR: electronic health record
HIPAA: Health Insurance Portability Accountability Act
PCM: principal care management
RPM: remote patient monitoring
UPDRS: Unified Parkinson Disease Rating Scale
